# Epidemiology of Sensitivity to Nickel, Cobalt and Chromium in Israel: A Retrospective Cohort Study

**DOI:** 10.1111/cod.14820

**Published:** 2025-06-03

**Authors:** Daniel Hilewitz, Akiva Trattner, Asher Hackett, Adi Raviv, Yehonatan Noyman, Stav Endelman, Dan Slodownik, Danny Daniely, Rivka Friedland, Daniel Mimouni, Igor Snast

**Affiliations:** ^1^ Department of Dermatology Rabin Medical Center–Beilinson Hospital Petach Tikva Israel; ^2^ Faculty of Medicine, Tel Aviv University Tel Aviv Israel; ^3^ Department of Dermatology Tel Aviv Sourasky Medical Center, Tel Tel Aviv Israel; ^4^ Pediatric Dermatology Unit Schneider Children's Medical Center of Israel Petach Tikva Israel

**Keywords:** allergic contact dermatitis, cross‐reactions, epidemiology, metals, patch test

## Abstract

**Background:**

Nickel, cobalt and chromium are three common allergens included in the European baseline series (EBS). It was shown that sensitivity to nickel and cobalt is more frequent among young women, most commonly due to contact with inexpensive jewellery, while sensitivity to chromium is more common among older men. Israel is located in West Asia and hosts the largest Jewish population in the world. There is limited data regarding the epidemiology of these metals in Israel or in the Mediterranean region.

**Objective:**

To investigate the epidemiology of sensitivity to nickel, cobalt and chromium in the EBS in a single center in Israel between 2009 and 2023.

**Methods:**

Retrospective cohort study that included all patients who underwent patch testing with the EBS in a tertiary center in Israel between 2009 and 2023.

**Results:**

Of 5234 consecutive patients (1679 males [32.1%]) 2158 (41%) were sensitive to nickel, 541 (10.3%) to cobalt and 383 (7.3%) to chromium. During the study period, nickel sensitivity was stable and was associated with female sex and age 18–40 years. Among both sexes, cobalt sensitivity decreased significantly from 11.7% in 2009–2011 to 7.9% in 2020–2023 and was associated with female sex and age < 18 years. Chromium sensitivity decreased significantly from 11.1% in 2009–2011 to 5% in 2020–2023 and was associated with male sex and older age (> 60 years). Among both sexes, cobalt strongly co‐reacted with nickel (OR = 1.69, 95% CI 1.38–2.06, *p* < 0.001) and chromium (OR = 3.57, 95% CI 2.67–4.55, *p* < 0.001). Nickel‐cobalt co‐sensitization was significantly more common among patients with strong (++) or very strong (+++) nickel sensitivity compared to patients with weak (+) sensitivity. Males, but not females, with nickel sensitivity positively co‐reacted with chromium. Among patients with nickel sensitivity, the strong (++) or very strong (+++) reaction (36.3%) was significantly more common compared to patients with cobalt or chromium sensitivity.

**Conclusion:**

In this retrospective Israeli study, the prevalence of sensitivity to nickel was stable but much higher compared to European and North American studies, highlighting the necessity for multicenter and general population studies and possibly a legislation regarding nickel restriction of items with prolonged contact with the skin. Cobalt and chromium sensitivities decreased and were in line with previous studies.

## Graphical abstract

This Israeli retrospective study found a consistently high prevalence of nickel sensitivity, exceeding rates reported in Europe and North America, underscoring the need for broader population studies. The findings may reflect differences in legislation on nickel exposure. In contrast, cobalt and chromium sensitivities declined, aligning with prior research.

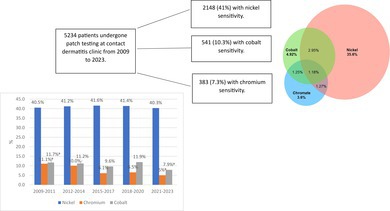

## 1 Introduction

Nickel, cobalt and chromium are the three metals included in the European baseline series (EBS). While all three metals are common contact allergens, nickel is the leading allergen detected by patch testing in most industrialised countries worldwide [1, 2].

Previous studies have shown the relative frequency of positive reactions to metals varies by geographic location, population, age and sex [3]. In a systematic review of general population studies, the pooled prevalences of contact allergy to nickel, cobalt and chromium were 11.4% (95% confidence interval: 9.4%–13.5%), 2.7% (2.1%–3.4%) and 1.8% (1.3%–2.6%), respectively, while in patients with dermatitis, prevalences are higher (nickel: 13.1%–30.3%, cobalt: 2%–19.6%, chromium: 0.9%–13.1%) [4, 5]. Sensitivity to nickel is more frequent among women and patients younger than 40 years of age, most commonly due to contact with consumer products such as inexpensive jewellery and metals in clothing [6], while sensitivity to chromium is more common among men and patients older than 40 years of age, most commonly due to either occupational exposure to the construction industry or consumer products such as leather accessories [7, 8, 9]. Similarly to nickel, but to a lesser degree, sensitivity to cobalt is more common among women and young patients; however, its relevance is frequently unclear and isolated sensitivity to cobalt is uncommon as it frequently co‐reacts with nickel and chromium [3].

In the United States and Canada, an increase in nickel allergy was reported, mostly attributed to the increasing frequency of nickel sensitivity among women [10, 11]. In contrast, in several European countries, an opposite trend of decreasing frequency of nickel sensitivity was recorded, most likely attributable to the implementation of nickel‐restrictive legislature [12].

Israel is a country in West Asia situated in the Middle East that shares borders with Lebanon, Syria, Jordan, Egypt and the Gaza strip. Israel hosts the largest Jewish population in the world, and Jews comprise Israel's largest ethnic and religious community. The current Israeli study was conducted in Rabin Medical Center, a part of Clalit Health Services, the largest health maintenance organisation in Israel and its largest tertiary center in Gush Dan, the largest metropolitan area in Israel (4.3 million population). In a previous study conducted in our center on 943 patients between 1999 and 2000, nickel, cobalt and chromium prevalences were 17.4%, 7.5% and 10.6%, respectively [13]. In 2006, Lazarov reported the results from an Israeli contact dermatitis clinic on 2156 patients between 1998 and 2004, reporting nickel, cobalt and chromium prevalences of 13.9%, 3.4% and 3.8%, respectively [14].

In a recent Israeli study (*n* = 2086, 2020–2022) Slodownik et al. [15] reported in a tertiary center in Tel‐Aviv nickel, cobalt and chromium sensitivity in the EBS of 14.3%, 2.8% and 1.2%, respectively.

As there is limited up to date data regarding the prevalence of sensitivity to nickel, cobalt and chromium in Israel or in the Mediterranean region, we aim in the current retrospective Israeli cohort study to examine the prevalence of sensitisation to these metals, with stratification of our results by sex and age over the years 2009 and 2023.

## 2 Materials and Methods

This retrospective study encompassed all patients referred to the contact dermatitis clinic at Rabin Medical Center, a tertiary hospital affiliated with Tel Aviv University, between 26 December 2009 and 28 December 2023. AT, a board certified dermatologist and a member of our group, was the head of the contact dermatitis clinic between 1995 and 2022, and during this time period, the routine of performance and reading of patch testing did not change. Outpatients medical referrals (almost exclusively by dermatologists or allergists) were screened by the head of the contact dermatitis clinic, and only cases where contact dermatitis was suspected underwent patch testing. All patients underwent patch testing at our center using the EBS (Chemotechnique Diagnostics, Malmo, Sweden) using Finn chambers on Scanpor tape (Hermal, Reinbeck, Germany). The allergens were applied to patches placed on unaffected skin of the upper back for 2 days. Readings were performed on days 2 and 3 according to the guidelines of the International Contact Dermatitis Research Group and scored as weak (+), strong (++), or very strong/extreme (+++). Clinical relevance was defined according to the ESCD criteria [16]. Both the performance and reading were performed by 4 well‐trained nurses during the entire study period. Subjects who had applied topical corticosteroids to test sites or received oral corticosteroids within 2 weeks before the study, as well as patients that received phototherapy 1 month prior to patch testing, were excluded [16]. Data regarding patients with positive patch tests to nickel sulphate hexahydrate 5% pet., cobalt chloride hexahydrate 1% pet. and potassium dichromate 0.5% pet. were extracted from the patch test database and electronic patient records. Data collected included sex, age and patch test results. Data regarding relevance, sources of exposure and occupation were not available in our electronic database. Thus, to improve the understanding of our findings, in a post hoc search we manually went over the patients between the years 2021 and 2023 and extracted the aforementioned data. In all, 99 patients (1.9%) completed patch tests more than once. In cases of discrepancy, the stronger reaction was chosen. Data prior to December 2009 was not computerised and thus was not available for analysis. This retrospective study was approved by the institutional ethics committee of Rabin Medical Center and registered with the Israel Ministry of Health.

### 2.1 Statistical Analysis

We present categorical variables with adjusted frequency, while continuous variables are reported as mean ± standard deviation. *T* test for independent groups was used to compare the mean differences between two independent groups. The association between two categorical variables was assessed by using the Chi‐square test or Fisher's exact test (two‐tailed). *p* value ≤ 0.05 was considered statistically significant.

## 3 Results

### 3.1 Study Population

Between April 2009 and end‐December 2023, a total of 5234 consecutive patients underwent patch testing with nickel, cobalt and chromium. Overall, 1679 were male (32.1%), with a mean age of 48.7 ± 20.9 years, while 3555 were female (67.9%), with a mean age of 43 ± 18.5 years. Of these patients, 391 (7.5%) were younger than 18 years of age (251 girls, 140 boys), with a mean age of 11.67 ± 4.64 years (Table 1). There were no significant demographic changes during the study period except a slightly lower percent of women and lower overall number of patch‐tested patients in years 2009–2011 compared to years 2012–2023 (Table S1).

**TABLE 1:** Sensitivity rates for nickel, cobalt and chromate by demographic characteristics.

Demographics	No. of patients, *n* (%)	OR (95% CI)	*p*
**T** **otal cohort**	**5234**		
Sex			
Male	1679 (32.1%)		
Female	3555 (67.9%)		
Age			
< 18 years	391 (7.5%)		
18–40 years	1895 (36.2%)		
40–60 years	1583 (30.2%)		
> 60 years	1365 (26.1%)		
**Nickel(II) sulphate hexahydrate 5.0% pet**	**2148 (41%)**		
Sex			
Males	289 (17.2%)	1.0 (reference)	
Females	1859 (52.3%)	5.272 (4.57–6.08)	< 0.0001
Age			
< 18 years	136 (34.8%)	0.57 (0.45–0.71)	< 0.0001
18–40 years	1043 (55%)	1.0 (reference)	
40–60 years	650 (41.1%)	0.74 (0.65–0.84)	< 0.001
> 60 years	319 (23.4%)	0.25 (0.21–0.29)	< 0.0001
**Cobalt(II) chloride hexahydrate 1.0% pet**	**541 (10.3%)**		
Sex			
Males	127 (7.5%)	1.0 (reference)	
Females	414 (11.6%)	1.61 (1.31–1.98)	< 0.001
Age			
< 18 years	65 (16.6%)	1.39 (1.03–1.87)	0.03
18–40 years	238 (12.5%)	1.0 (reference)	
40–60 years	155 (9.8%)	0.76 (0.61–0.94)	0.01
> 60 years	83 (6.1%)	0.45 (0.35–0.58)	< 0.0001
**Chromium 0.5% pet**	**383 (7.3%)**		
Sex			
Males	163 (9.7%)	1.0 (reference)	
Females	220 (6.2%)	0.61 (0.5–0.76)	< 0.001
Age			
< 18 years	33 (8.4%)	1.43 (0.95–2.16)	0.09
18–40 years	96 (5.1%)	1.0 (reference)	
40–60 years	124 (7.8%)	1.32 (1.0–1.74)	0.05
> 60 years	130 (9.5%)	1.64 (1.24–2.15)	< 0.001

### 3.2 Patch Test Results

**FIGURE 1:**
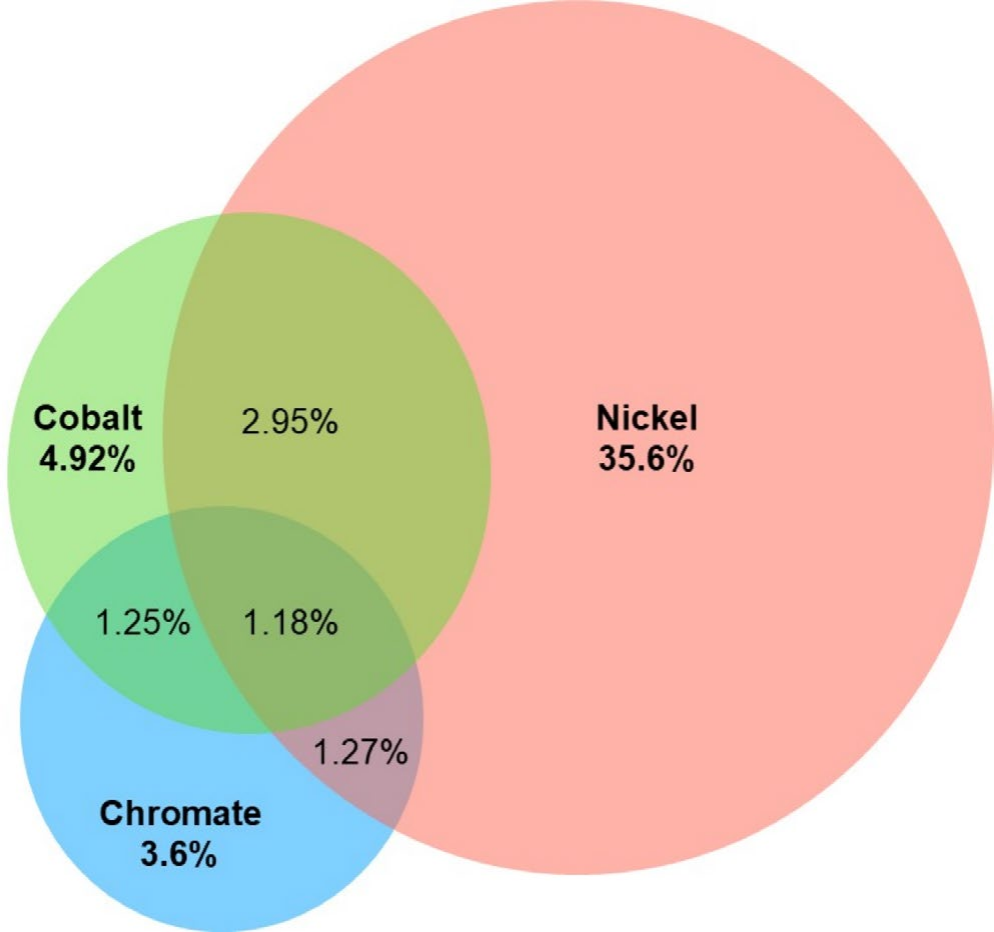
Venn diagram demonstrating the percentage of overall study population found with allergy to nickel sulphate, cobalt and chromate.

Of 5234 patch tested patients, 2148 (41%) were sensitive to nickel, 541 (10.3%) were sensitive to cobalt and 383 (7.3%) were sensitive to chromium. Overall, 2053 (39.2%) patients reacted to only 1 metal, 412 (7.9%) to 2 metals, 65 (1.2%) to 3 metals and 2704 (51.6%) tested negative to all metals (Table 2). Females compared to males reacted significantly more frequently (*p* < 0.001) to one or two metals, while reactivity to all 3 metals was comparable between sexes. Overall, 35.6% had isolated nickel sensitivity (86.8% of patients with nickel sensitivity), significantly higher compared to 4.92% with isolated cobalt sensitivity (47.8% of patients with cobalt sensitivity) and 3.6% with isolated chromium sensitivity (49.3% of patients with chromium sensitivity) (*p* < 0.001) (Figure 1).

**TABLE 2:** Sensitivity to one, two or three metals in males and females.

Sex	No sensitivity	Sensitivity to 1 metal	Sensitivity to 2 metals	Sensitivity to 3 metals
Males (*n* = 1679)	1217 (72.5%)^a^	360 (21.4%)^a^	87 (5.2%)^a^	15 (0.9%)
Females (*n* = 3555)	1487 (41.8%)^a^	1693 (47.6%)^a^	325 (9.1%)^a^	50 (1.4%)
Overall (*N* = 5234)	2704 (51.6%)	2053 (39.2%)	412 (7.9%)	65 (1.2%)

^a^ *p* < 0.05: *χ* ^2^ test comparing sensitivity to number of metals within same sex category.

Among both sexes cobalt strongly co‐reacted with nickel (OR_total_ = 1.69, 95% CI 1.38–2.06, *p* < 0.001) and chromium (OR_total_ = 3.57, 95% CI 2.67–4.55, *p* < 0.001) (Table 3). Patients with strong (++) or very strong (+++) nickel sensitivity compared to patients with weak (+) sensitivity were more likely to co‐react with cobalt (OR_total_ = 2.23, 95% CI 1.77–2.82, *p* < 0.0001) (Table 4). Males with nickel sensitivity positively co‐reacted with chromium (OR_males_ = 1.92, 95% CI 1.32–2.78, *p* < 0.001), while females with nickel sensitivity negatively co‐reacted with chromium (OR_females_ = 0.63, 95% CI 0.47–0.82, *p* < 0.001) (Table 3).

**TABLE 3:** Double sensitisation of nickel cobalt and chromium.

		No nickel sensitivity	Nickel sensitivity
Chromium sensitivity	Males	119/1390^a^ (8.5%)	44/289^a^ (15.2%)
	Females	129/1696^a^ (7.6%)	91/1859^a^ (5%)
	Overall	248/3086^a^ (8.04%)	135/2148^a^ (6.28%)
Cobalt sensitivity	Males	83/1390^a^ (6%)	44/289^a^ (15.2%)
	Females	120/1696^a^ (7.1%)	294/1859^a^ (15.8%)
	Overall	203/3086^a^ (6.58%)	228/2148^a^ (15.73%)

		No cobalt sensitivity	Cobalt sensitivity
Chromium sensitivity	Males	119/1552^a^ (7.6%)	44/127^a^ (34.6%)
	Females	130/3141^a^ (4.1%)	90/414^a^ (21.7%)
	Overall	249/4693^a^ (5.3%)	134/541^a^ (24.7%)

^a^ *p* < 0.05: *χ* ^2^ test comparing males, females and overall population.

**TABLE 4:** Association between strength of reaction to nickel and sensitivity to cobalt and chromium.

	Nickel sensitivity		
	+ (Mild) (*n* = 1368)	++ (Strong) (*n* = 712)	+++ (Very strong) (*n* = 68)
Chromium sensitivity	89 (6.5%)	39 (5.5%)	7 (10.3%)
Cobalt sensitivity	160 (11.7%)^a^	147 (20.6%)^a^	31 (45.6%)^a^

^a^ *p* < 0.05: *χ* ^2^ test comparing nickel sensitivity of mild versus strong‐to‐very strong response.

### 3.3 Trends and Associations With Sex and Age

#### 3.3.1 Nickel

**FIGURE 2:**
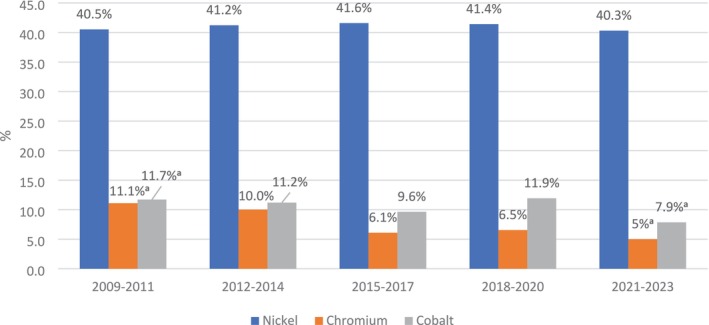
Prevalence of sensitivity to nick, cobalt and chromium among 5532 patients between 2009 and 2023. ^a^ *p* < 0.05: *χ* ^2^ test.

During the study period, the overall frequency of nickel sensitivity was stable with non‐significant fluctuations (Figure 2) and with a mean prevalence of 41%. Stratification by sex revealed a statistically significant decrease in frequency among males from 21.4% in 2009–2011 to 15% in 2020–2023 (*p* < 0.05). Trend analysis by age revealed a decrease in nickel sensitivity only among males < 60 years (*p* = 0.03), while prevalence among older men and women was stable (Table 5) Patients with nickel sensitivity were significantly more likely to be women (OR_total_ = 5.27, 95% CI 4.57–6.08, *p* < 0.001) and aged 18–40 years, with a graded decrease in sensitivity frequency commencing from the 18–40 years group (55%) to the > 60 years group (23.4%) (Table 1).

**TABLE 5:** Sensitivity to nickel by study period in males and females.

Years	Men < 40 *n*/*N*, (%)	Men 40–60 *n*/*N*, (%)	Men > 60 *n*/*N*, (%)	Overall men *n*/*N* (%)	Women < 40 *n*/*N*, (%)	Women 40–60 *n*/*N* (%)	Women > 60 *n*/*N* (%)	Overall women *n*/*N*, (%)
2009–2011	27/96 (28.1)^a^	17/81 (21)^a^	9/71 (12.7)	53/248 (21.4)	130/205 (63.4)	60/121 (49.6)	20/75 (26.7)	210/401 (52.4)
2012–2014	33/140 (23.6)	16/99 (16.2)	15/112 (13.4)	64/351 (18.2)	193/312 (61.9)	122/219 (55.7)	37/127 (29.1)	352/658 (53.5)
2015–2017	25/107 (23.4)	11/76 (14.5)	10/121 (8.3)	46/304 (15.1)	193/318 (60.7)	130/229 (56.8)	54/166 (32.5)	377/713 (52.8)
2018–2020	28/141 (19.9)	21/122 (17.2)	23/154 (14.9)	72/417 (17.3)	294/440 (66.8)	116/249 (46.6)	36/193 (29)	466/882 (52.8)
2021–2023	21/112 (18.8)^a^	14/107 (13.1)^a^	19/140 (13.6)	54/359 (15)	235/415 (56.6)	143/280 (51.1)	76/206 (36.9)	454/901 (50.4)

*Note: n*, indicates number of patients with metal positivity by sex and study period. *N* indicates overall number of patch‐tested patients by sex and study period.

^a^ Due to small sample size, a statistically significant decrease in nickel sensitivity among males during the study period was evident only when the < 40 and 40–60 years groups were combined.

#### 3.3.2 Cobalt

During the study period, cobalt sensitivity decreased significantly from 11.7% in 2009–2011 to 7.9% in 2020–2023 (*p* < 0.05) (Figure 2). This trend was statistically significant across both sexes; however, it did not reach statistical significance among males due to small sample size (Table 6). Patients with cobalt sensitivity were significantly more likely to be women (OR_total_ = 1.61, 95% CI 1.31–1.98, *p* < 0.001) and aged < 18 years (16.6%) with a graded decrease in sensitivity to those > 60 years (6.1%) (Table 1).

**TABLE 6:** Sensitivity to nickel, cobalt and chromate in males and females over the study period.

Years	Nickel sulphate, *n*/*N* (%)		Cobalt chloride, *n*/*N* (%)		Potassium dichromate, *n*/*N* (%)	
	Males	Females	Males	Females	Males	Females
2009–2011	53/248 (21.4)^a^	210/401 (52.4)	20/248 (8.1)	56/401 (13.9)^a^	33/248 (13.3)^a^	39/401 (9.7)^a^
2012–2014	64/351 (18.2)	352/658 (53.5)	30/351 (8.5)	83/658 (12.6)	44/351 (12.5)	57/658 (8.6)
2015–2017	46/304 (15.1)	377/713 (52.8)	23/304 (7.5)	75/713 (10.5)	23/304 (7.5)	39/713 (5.5)
2018–2020	72/417 (17.3)	466/882 (52.8)	37/417 (8.8)	118/882 (13.4)	36/417 (8.6)	49/882 (5.5)
2021–2023	54/359 (15)^a^	454/901 (50.4)	17/359 (4.7)	82/901 (9.1)^a^	27/359 (7.5)^a^	36/901 (4)^a^

*Note: n*, indicates number of patients with metal positivity by sex and study period. *N* indicates overall number of patch‐tested patients by sex and study period.

^a^ *p* < 0.05: *χ* ^2^ test comparing males and females.

#### 3.3.3 Chromium

During the study period, chromium sensitivity decreased significantly from 11.1% in 2009–2011 to 5% in 2020–2023 (*p* < 0.05) (Figure 2). This trend was statistically significant across both sexes. Patients with chromium sensitivity were significantly more likely to be males (OR = 1.63, 95% CI 1.31–2, *p* < 0.001) and aged > 60 years (Table 6).

### 3.4 Strength of Reaction

Of 2148 patients with nickel sensitivity, in 780 (36.3%) strength of reaction was strong (++) or very strong (+++), significantly higher compared to 14.8% (80/541) of patients with cobalt sensitivity (OR = 3.29, 95% CI 2.55–4.23, *p* < 0.0001) and to 19.6% (75/383) of patients with chromium sensitivity (OR = 2.34, 95% CI 1.79–3.06, *p* < 0.0001) (Table 7), and was significantly higher among females compared to males (OR = 1.38, 95% CI 1.06–1.8, *p* = 0.02), and among patients aged < 40 years or 40–60 years compared to patients aged > 60 years (OR = 2.68, 95% CI 2–3.59, *p* < 0.0001). In contrast, among patients with cobalt or chromium sensitivity, no association was found between sex or age and strength of reaction.

**TABLE 7:** Association of strength of reaction to nickel, cobalt and chromate with sex and age.

Metal	Sex, age (year)	Mild (+)	Strong (++)	Very strong (+++)
Nickel sulphate				
Sex	Male (*n* = 289)	202 (69.9%)	75 (25.9%)^a^	12 (4.1%)^a^
	Female (*n* = 1859)	1166 (62.7%)	637 (34.3%)^a^	56 (3%)^a^
Age	< 40 years (*n* = 1179)	696 (59%)	435 (36.9%)^b^	48 (4.1%)^b^
	40–60 years (*n* = 650)	415 (63.8%)	218 (33.5%)^b^	17 (2.6%)^b^
	> 60 years (*n* = 319)	257 (80.5%)	59 (18.5%)^b^	3 (0.9%)^b^
Overall	Overall (*N* = 2148)	1368 (63.7%)	712 (33.1%)^c^	68 (3.2%)^c^
Cobalt chloride				
Sex	Male (*n* = 127)	107 (84.2%)	18 (14.2%)	2 (1.6%)
	Female (*n* = 414)	354 (85.5%)	55 (13.3%)	5 (1.2%)
Age	< 40 (*n* = 303)	257 (84.8%)	41 (13.5%)	5 (1.6%)
	40–60 (*n* = 155)	131 (84.5%)	23 (14.8%)	1 (0.6%)
	> 60 (*n* = 83)	73 (87.9%)	9 (10.8%)	1 (1.2%)
Overall	Overall (*N* = 541)	461 (85.2%)	73 (13.5%)^c^	7 (1.3%)^c^
Potassium dichromate				
Sex	Male (*n* = 163)	130 (79.7%)	31 (19%)	2 (1.2%)
	Female (*n* = 220)	178 (80.9%)	38 (17.3%)	4 (1.8%)
Age	< 40 (*n* = 129)	98 (76%)	27 (20.9%)	4 (3.1%)
	40–60 (*n* = 124)	100 (80.7%)	24 (19.3%)	0 (0%)
	> 60 (*n* = 130)	110 (84.6%)	18 (13.8%)	2 (1.5%)
Overall	Overall (*N* = 383)	308 (80.4%)	69^b^ (18%)	6^b^ (1.6%)

^a^ *p* < 0.05: *χ* ^2^ test comparing males and females among patients with nickel sensitivity (mild vs. strong or very strong response).

^b^ *p* < 0.05: *χ* ^2^ test comparing age < 40 or 40–60 years with age > 60 years among patients with nickel sensitivity (mild vs. strong or very strong response).

^c^ *p* < 0.05: *χ* ^2^ test comparing total population of nickel‐sensitivity with cobalt‐ and chromate‐sensitive patients in terms of strong or very strong response.

### 3.5 Relevance, Sources of Exposure and Occupational Data (2021–2023)

Between the years 2021 and 2023 there were 508, 99 and 62 patients diagnosed with nickel, cobalt and chromium sensitivity, respectively (Table 8). Of patients in whom data was available, 53% had a relevant reaction to nickel, 48% to cobalt and 55% to chromium (*p* > 0.05). A higher proportion of patients with nickel sensitivity (25.4%) had a past relevant reaction compared to patients with cobalt (12.5%) or chromium (6.1%) sensitivity. The most frequent identified source of nickel (71.9%) and cobalt (50%) sensitivity was jewellery, while the most common source of chromium sensitivity was leather‐related accessories including tefillin (43.5%) and shoes (26.1%). In the majority of patients with nickel (95.1%), cobalt (85.7%) and chromium (88.6%) sensitivity, the exposure was non‐occupational.

**TABLE 8:** Relevance, sources of exposure and occupational data (2021–2023).

		Nickel sulphate (*N* = 508)	Cobalt chloride (*N* = 99)	Chromium (*N* = 62)
Relevance	Available data	346 (68.1%)	64 (64.6%)	49 (79%)
	Relevant	184 (53%)	31 (48%)	27 (55%)
	Current	96 (27.7%)	23 (36%)	24 (49%)
	Past	88 (25.4%)	8 (12.5%)	3 (6.1%)
	Not relevant	162 (46.8%)	33 (33.3%)	22 (44.9%)
Occupational	Available data	228 (44.9%)	49 (49.4%)	35 (56.4%)
	Occupational	11 (4.8%)	7 (14.3%)	4 (11.4%)
Sources	Available data	82 (16.7%)	18 (18.2%)	23 (37.1%)
	Jewellery	59 (71.9%)	9 (50%)	2 (8.7%)
	Watch	0 (0%)	0 (0%)	0 (0%)
	Belt	4 (4.9%)	2 (11.1%)	0 (0%)
	Clothing	1 (1.1%)	0 (0%)	0 (0%)
	Shoes	1 (1.1%)	2 (11.1%)	6 (26.1%)
	Makeup	2 (2.4%)	2 (11.1%)	0 (0%)
	Hair dye	3 (3.6%)	2 (11.1%)	0 (0%)
	Razor	2 (2.4%)	1 (5.5%)	1 (4.3%)
	Cement	0 (0%)	0 (0%)	1 (4.3%)
	Tefillin	0 (0%)	0 (0%)	10 (43.5%)
	Other	18 (21.9%)	8 (44.4%)	13 (56.5%)

## 4 Discussion

This retrospective cohort study examined 5234 patch tested patients for nickel, cobalt and chromium sensitivity during a 15‐years period in a tertiary Israeli contact dermatitis clinic.

In our previous study (*N* = 943, 1999–2000) nickel, cobalt and chromium prevalences were 17.4%, 7.5% and 10.6%, respectively [13]. Thus, the main finding of our study is that between 2000 and 2009 (time period between previous study and current study) sensitivity of nickel increased significantly from 17.4% to 41%. Additionally, sensitivity of cobalt increased from 7.5% in 1999–2000 to 11.7% in 2009–2011 and decreased back to 7.9% in 2021–2023, while sensitivity of chromium remained stable from 1999–2000 to 2009–2011 (11.1%) and then decreased to 5% in 2021–2023.

Nickel prevalence in our study is higher than reported values from North America and Europe, while cobalt and chromium prevalences fell within the wide range of reported values [5]. There is a significant variability between studies regarding metal sensitivity attributable to differential selectivity for patch testing, variance in nickel and chromium regulation implantation and socioeconomic and environmental factors. In accordance with our finding in the study by the North American Contact Dermatitis Group (NACDG) (*n* = 44 097) conducted between 1994 and 2014, a significant increase in nickel sensitivity was reported from 14.3% to 20.1%, attributable to increased exposure to nickel‐releasing metal objects such as jewellery and electronics [11]. In contrast, in 1991, Danish legislation limited the amount of nickel released from items that may come into direct and prolonged contact with the skin. The European Nickel Directive took effect in 2000 and was amended in 2004 to include piercing post assemblies and was further incorporated into the European regulations in 2006. Consequently, nickel prevalence decreased in several European countries including Germany, the United Kingdom, Italy, Sweden and Denmark [12]. In a multi‐center European study across 13 countries (*n* = 7675, 2019–2020) by the European Surveillance System in Contact Allergies (ESSCA) and European Society of Contact Dermatitis (ESCD) reported nickel, cobalt and chromium prevalences of 19.8%, 6.2% and 4.4%, respectively, were reported; however, prevalences varied significantly, being highest in Austria and lowest in the United Kingdom (nickel: 30.3% vs. 13.1%, cobalt: 19.6% vs. 2%, chromium: 13.1% vs. 0.9%) [5].

As the relevance of patch test results was not available in our electronic database, to improve the understanding of our findings we made a post hoc manual search between the years 2021 and 2023, finding a clinical relevance (past or present) of 53% among patients reactive to nickel, 48% to cobalt and 55% to chromium, which falls within the wide range of reported values. For instance, in a recent prospective multicentric study of the Spanish Contact Dermatitis Register (REDIAC) across different age groups, a relevance ranging between 5.4% to 23.3% was reported for nickel [17], whereas in the NACDG study 12.7% and 47.5% of patients had current (definite or probable) or past reactions to nickel, 5% and 36.9% to cobalt and 21.3% and 18% to chromium [9].

We believe the high nickel prevalence may be attributable to several factors. First, in Israel, there is no legislation regarding nickel restriction. Second, imports of consumer products from China have significantly increased over the past two decades and, according to the Israel Central Bureau of Statistics, China became Israel's largest source of imports in 2021, surpassing the U.S., with significant increase in online purchase from ‘Ali Express’ and ‘Shein’, where Israeli spent the most money among the foreign‐based shopping sites in 2022 [18, 19]. As this purchased merchandise include cheap metallic items such as jewellery, electronics and toys containing nickel it would be expected that in accordance with our findings nickel sensitivity in Israel would be high [19]. This is further supported by the finding in our study that for the vast majority of patients (> 95%) with nickel sensitivity the exposure was non‐occupational and among patients with relevant reaction jewellery was the most common source of exposure (71%) slightly higher compared to NACDG study (60%) [9]. Nonetheless, in a recent Israeli study (*n* = 2086, 2020–2022) Slodownik et al. [15] reported in a tertiary center in Tel‐Aviv nickel, cobalt and chromium sensitivity in the EBS of 14.3%, 2.8%, 1.2%, respectively. Similarly, the HEMA prevalence rate in our previous study was significantly higher compared to Slodownik et al. (8.1% vs. 2.6%) [20]. The significant and consistent disparity of results may be attributed at least partially to differential patients' selectivity. In our center, we screen outpatients referrals and we do not perform patch tests in cases where testing is judged to be of low yield (for instance pruritus without primary dermatosis). Additionally, our center is part of Clalit Health Services, the largest health maintenance organisation in Israel and its largest tertiary center in Gush Dan, the largest metropolitan area in Israel (4.3 million population). Thus, our catchment population is likely similar or higher, yet our clinic performs significantly fewer patch tests per year (~430 vs. 700) and it was shown that as the intensity of patch testing increases, the frequency of positive reactions decreases [21]. It should be noted that, although at both centers the performance of patch testing and timeline of reading is similar, in our clinic, well‐trained nurses perform the reading while at the other centre reading is performed by dermatologists associated with the contact dermatitis clinic. It may be claimed that irritant reactions may have been interpreted more frequently as positive by nurses in our center; however, it is accepted that well‐trained nurses are fit to perform patch test reading and this is a common practise in many centres [22]. Additionally, it was shown that nickel had significantly fewer irritant reactions compared to cobalt and chromium. For instance, in a study of 853 hard metal workers, non‐allergic reactions appeared in 6.5% of the nickel tests compared to 13% of the chromium tests and 18.3% of the cobalt tests [23]. As both cobalt and chromium decreased significantly during the study period and the nursing staff did not change during the study period, it seems unlikely that false‐positive reading of nickel reactions had a significant influence on our results.

Although multicentre studies are needed to clarify the true prevalence of nickel in Israel, given the significant increase in nickel prevalence compared to our previous study, the findings of this study suggest that nickel regulating legislature may be required in Israel.

Interestingly, although the overall prevalence of nickel in this study was stable, in the subgroup of males younger than 60 years of age, a statistically significant trend of decreasing sensitivity to nickel was evident during the study period. This finding raises a question of whether there is decreased exposure among younger males to nickel‐containing products such as piercing and jewellery including watches.

The decrease of chromium sensitivity in our study is likely due to the Israeli legislation from the yearly 1990s that recommended lowering chromium concentration in consumer products including detergents and bleaches to lower than 5 ppm [24]. Accordingly, a previous Israeli study reported a decrease in the frequency of relevant exposure to chromate in detergent from 25% in 1999–2001 to 5.7% in 2011–2013 [24]. Of note, although exposure to chromium is frequently occupational due to its high prevalence in the construction industry, only in a single patient between the years 2021 and 2023 was the source of exposure cement. This may be attributable to the fact that the majority of construction workers in central Israel are non‐citizens and thus have less access to medical services such as patch testing. Interestingly, the most common source of exposure to chromium in our study was Tefillin, a religious article made of leather and worn by observant Jewish men during morning prayers on weekdays [25].

In accordance with previous studies, we found that females and young individuals were more likely to be sensitised to nickel and cobalt; however, sensitivity to cobalt was most common among children, while nickel sensitivity was common among young adults aged 18–40 years. In accordance with our findings, several North American studies found higher prevalence rates of sensitivity to cobalt among children compared to adults. In the NACDG study (2001–2018) the prevalence of cobalt among children aged 0–17 years was 11.8% (*n* = 1110) compared to the prevalence of 7.1% among adults (*n* = 27 100) [26]. Nonetheless, there is limited data regarding sources of exposure to cobalt allergy in children compared to adults [27]. In contrast to nickel and cobalt, individuals with sensitivity to chromium were more likely to be males and older adults (> 60 years). The epidemiological disparity between chromium versus nickel and cobalt is reflected in the finding that females were more likely to be sensitive to 2 types of metals, while sensitivity to 3 types of metals was similar between the sexes. In accordance with previous studies, patients with nickel and chromium sensitivities were often co‐sensitised to cobalt, but co‐sensitisation of nickel and chromium was only evident among men in our study [3].

This is the first study to compare the strength of reaction between the three metals, finding that the strong [++] or very strong [+++] reaction to nickel (36.3%) was significantly more common compared to cobalt (14.8%) and chromium (19.6%), and within the group of patients with nickel sensitivity, the strong or very strong reaction was associated with young age and female sex. This may be attributable to the nature of sensitisation of young women to nickel involving prolonged contact with jewellery, whereas among men and older ages, exposure to nickel is likely less intense and waning immunity further decreases the degree of sensitisation at older ages [28]. Similar to our finding, a Canadian study reported that 39.7% of patients with nickel sensitivity had a strong or very strong reaction; however, comparison to other metals was not performed [10].

Nickel has been hypothesised to potentiate the sensitization to cobalt, given the concurrent co‐sensitisation [29]. This theory is supported by our finding that nickel and cobalt co‐sensitization was significantly more common among patients with strong or very strong sensitivity to nickel compared to patients with a weak sensitivity. Interestingly, nickel and chromium co‐sensitisation was evident only among men, possibly reflecting occupational exposure to both metals, including in the construction setting.

In conclusion, in this retrospective Israeli study, the prevalence of sensitivity to nickel was stable but much higher compared to European and North American studies and our previous study. In more than half of cases, reactions to nickel were relevant and jewellery was by far the most common source of exposure. To improve the understanding of our results, multicenter and general population studies are needed as well as market surveys evaluating prevalences of nickel‐releasing items, which might highlight the necessity for a legislation regarding nickel restriction of items intended to come into direct and prolonged contact with the skin. Sensitivity to cobalt and chromium decreased during the study period and was in line with previous studies, as was the pattern of co‐sensitization between cobalt‐nickel and cobalt‐chromium.

## Author Contributions

**Daniel Hilewitz:** writing – original draft. **Akiva Trattner:** writing – original draft. **Asher Hackett:** writing – original draft. **Adi Raviv:** writing – original draft. **Yehonatan Noyman:** writing – original draft. **Stav Endelman:** writing – original draft. **Dan Slodownik:** writing – original draft. **Danny Daniely:** writing – original draft. **Rivka Friedland:** writing – original draft. **Daniel Mimouni:** writing – original draft. **Igor Snast:** writing – original draft, conceptualization.

## Ethics Statement

Reviewed and approved by the Ethics Committee of Rabin Medical Center.

## Consent

The authors have nothing to report.

## Conflicts of Interest

The authors declare no conflicts of interest.

## Supporting information


**Data S1.** cod14820‐sup‐0001‐supinfo.

## Data Availability

The data that support the findings of this study are available from the corresponding author upon reasonable request.
