# Supplementary material for: Epidemiology of Sensitivity to Nickel, Cobalt and Chromium in Israel: A Retrospective Cohort Study
Source: Contact Dermatitis. 2025 Jun 3;93(3):204–13. doi: 10.1111/cod.14820 (PMC12318911; doi:10.1111/cod.14820)
Supplement: Supplementary file 1 — Data S1. cod14820‐sup‐0001‐supinfo. [file COD-93-204-s001.docx]

**Supplementary data**

Epidemiology of Sensitivity to Nickel, Cobalt and Chromium in Israel : A Retrospective Cohort Study

Table 1s. Demographics of the overall population during the study period 2009-2023.

| Mean age (years) | Above 40 years | Female | Male | Total patients |  |
| --- | --- | --- | --- | --- | --- |
| $43.37\pm19.33$ | 348 (53.6%) | 401 (61.8%) | 248 (38.2%) | 649 | 2009-2011 |
| $44.03\pm19.28$ | 557 (55.2%) | 658 (65.2%) | 351 (34.8%) | 1009 | 2012-2014 |
| $45.49\pm19.41$ | 592 (58.2%) | 713 (70.1%) | 304 (29.9%) | 1017 | 2015-2017 |
| $44.73\pm19.93$ | 718 (55.3%) | 882 (67.9%) | 417 (32.1%) | 1299 | 2018-2020 |
| $45.73\pm19.35$ | 733 (58.2%) | 901 (71.5%) | 359 (28.49%) | 1260 | 2021-2023 |
